# Characterization of spinal cord tissue-derived extracellular vesicles in neuroinflammation

**DOI:** 10.1186/s12974-024-03147-y

**Published:** 2024-06-08

**Authors:** Larissa Jank, Ajay Kesharwani, Taekyung Ryu, Deepika Joshi, Dimitrios C. Ladakis, Matthew D. Smith, Saumitra Singh, Tanina Arab, Kenneth W. Witwer, Peter A. Calabresi, Chan-Hyun Na, Pavan Bhargava

**Affiliations:** 1 Department of Neurology, Johns Hopkins University School of Medicine, Baltimore, MD USA; 2 Institute for Cell Engineering, Johns Hopkins University School of Medicine, Baltimore, MD USA; 3 Department of Molecular and Comparative Pathobiology, Johns Hopkins University School of Medicine, Baltimore, MD USA; 4 Solomon H. Snyder Department of Neuroscience, Johns Hopkins University School of Medicine, Baltimore, MD USA

**Keywords:** Extracellular vesicles, EAE, Multiple sclerosis, Neuroinflammation, Synaptic pathology, Myelin, Tissue-derived extracellular vesicles

## Abstract

Extracellular vesicles (EVs) are released by all cells, can cross the blood–brain barrier, and have been shown to play an important role in cellular communication, substance shuttling, and immune modulation. In recent years EVs have shifted into focus in multiple sclerosis (MS) research as potential plasma biomarkers and therapeutic vehicles. Yet little is known about the disease-associated changes in EVs in the central nervous system (CNS). To address this gap, we characterized the physical and proteomic changes of mouse spinal cord-derived EVs before and at 16 and 25 days after the induction of experimental autoimmune encephalomyelitis (EAE), a neuroinflammatory model of MS. Using various bioinformatic tools, we found changes in inflammatory, glial, and synaptic proteins and pathways, as well as a shift in the predicted contribution of immune and glial cell types over time. These results show that EVs provide snapshots of crucial disease processes such as CNS-compartmentalized inflammation, re/de-myelination, and synaptic pathology, and might also mediate these processes. Additionally, inflammatory plasma EV biomarkers previously identified in people with MS were also altered in EAE spinal cord EVs, suggesting commonalities of EV-related pathological processes during EAE and MS and overlap of EV proteomic changes between CNS and circulating EVs.

**Supplementary Information:**

The online version contains supplementary material available at 10.1186/s12974-024-03147-y.

## Introduction

Multiple sclerosis (MS) is a chronic demyelinating disease of the central nervous system (CNS). Despite being the most frequent non-traumatic disabling neurological disease among young adults, the cause of MS and methods to predict its progression are still under investigation [1]. The widely-used experimental autoimmune encephalomyelitis (EAE) model of MS is a neuroinflammatory model where myelin-reactive T-cells, primed by injection of myelin peptide with an adjuvant, target oligodendrocytes, leading to neuroinflammation, particularly the spinal cord and anterior visual pathway [2].

Extracellular vesicles (EVs) are a heterogeneous group of membrane-delimited particles, with the most abundant “small EVs” often defined as 30–150 nm in diameter [3]. Released by all cells in the body, EVs are thought to cross the blood–brain barrier (BBB) bidirectionally [4]. Since EVs have many functions in health and disease, including communication, substance shuttling, and immune modulation [5, 6], they have provoked interest in neuroimmunology and MS research, where they are now being studied to better understand disease pathology, as a source of biomarkers, and as therapeutic vehicles [7–10]. In EAE and other demyelination models, EVs reportedly mediate antigen presentation, propagate inflammatory signals, resolve inflammation, induce tolerance, and promote remyelination [7, 10–14]. The relevance of EVs in EAE pathology, and hence also the importance of efforts to characterize them, is emphasized by the fact that A-SMase mice, which have impaired EV release through membrane budding, are highly resistant to EAE induction [15].

Currently, there is a lack of studies characterizing tissue-derived EVs in EAE, or other MS models. This is needed to understand what EV-mediated cellular communication is taking place during the disease process, in order to identify what changes to look for in biomarker studies. Ultimately understanding EV contents in disease might also help develop better EV-based therapeutic approaches.

Given this knowledge gap, we characterized EVs derived from the whole spinal cords of EAE mice. After first assessing the purity and physical characteristics of separated tissue EVs, we profiled EV proteins of naïve mice and mice at 16 and 25 days of EAE induction using 18-plex Tandem Mass Tags (TMT) proteomics. Data were used to identify molecular pathways and cell types contributing to the EV pool. We also compared our results with previously published peripheral EV biomarkers in MS [8, 9, 16–19]. We further compared the EV-enriched fractions with an intermediate separation product, the 10k pellet. In summary, our novel resource identifies 7000 proteins in CNS-derived EVs and catalogs changes during the course of EAE.

## Material and methods

### Mice

C57BL/6J mice were purchased from The Jackson Laboratory. All mice were kept in a pathogen-free animal facility at Johns Hopkins University School of Medicine, given standard food and water ad libitum, and subjected to a 12-h light/dark cycle. Use of experimental animals adhered strictly to National Institutes of Health guidelines, and all experimental protocols were approved by the Johns Hopkins Institutional Animal Care and Use Committee.

### Induction of EAE and clinical scoring

EAE was induced in 10–13-week-old female C57BL/6J mice as previously described [20]. In brief, to induce EAE, we subcutaneously injected 150 µg of MOG_33–55_ (obtained from Johns Hopkins Peptide Synthesis Core) along with complete Freund’s adjuvant (CFA) (ThermoScientific, Rockford, IL, USA) containing 600 μg of mycobacterium tuberculosis (BD, Franklin Lakes, NJ) in the lateral abdomen, followed by intraperitoneal injection with 300 ng of pertussis toxin (List Biological Labs, Campbell, California, USA) on the same day of immunization and after 2 days. EAE clinical scores were obtained daily in a blinded manner using the established standard clinical scoring system from 1 to 5: 0 = no signs of disease; 1 = loss of tail tonicity; 2 = loss of tail tonicity and mild paralysis of hindlimbs; 3 = paralysis of hindlimbs; 4 = hindlimbs paralysis and mild paralysis of forelimbs; 5 = complete paralysis or death.

### Tissue collection and processing

Mice were sacrificed either on day 16 post-immunization, corresponding to the peak stage of EAE, or on day 25, representing the chronic stage of EAE. After anesthesia with isoflurane and trans-cardiac perfusion with ice-cold PBS, whole spinal cords were collected and snap-frozen on dry ice before storage at − 80 °C. EVs were separated from frozen spinal cords.

### EV separation by size exclusion chromatography and ultracentrifugation

We separated EVs from mice spinal cords by size exclusion chromatography (SEC) and ultracentrifugation as described previously [21] with some modifications. Briefly, five frozen spinal cords (approximately 400 mg) from EAE mice (peak or chronic) or naïve mice were gently chopped into 2–4 mm pieces on dry ice and transferred to a 15 mL tube containing 75 U/mL Collagenase type III (Worthington #CLS-3) in Hibernate-E medium (Thermo #A12476-01) at a ratio of 800ul buffer (75 U/mL collagenase in Hibernate-E medium) per 100 mg of tissue, and incubated for 15 min at 37 °C. During this incubation, the tube was mixed every 5 min by inverting and pipetting up and down gently using a 10 mL pipette. After incubation, the tube was immediately transferred onto ice. Complete protease inhibitor (PI, Sigma #11697498001) and PhosSTOP (PS, Sigma #4906837001) solution were then added to stop the digestion. The dissociated tissue was centrifuged at 300×*g* for 10 min at 4 °C. The supernatant was collected and centrifuged at 2000×*g* for 15 min at 4 °C to remove remaining cell debris. The cell-free supernatant was then gently filtered (0.22 µm syringe filter, Millipore Sigma, SLGS033SS) to further deplete cell debris and centrifuged at 10,000×*g* for 30 min at 4 °C (S52-ST rotor, Beckman Ultra-Clear Tube with 5 mL capacity #344057). The pellet (“10k pellet”) was then resuspended in PBS. The supernatant containing small EVs was concentrated from 5 to 0.5 mL using a 100 kilodalton (kDa) molecular weight cut-off protein concentrator. (Millipore UFC901024). The resulting concentrate was applied onto a qEV Original 70 nm SEC column (IZON Science SP1-USD, Christchurch, New Zealand) and processed per manufacturer guidelines. After loading the sample, 0.5 mL fractions were collected by elution with PBS. The initial 3 mL (F1–6) eluate was designated as the void volume. Following this, a total of 2 mL eluate (Fractions 7–10) was collected and combined as EV-enriched fractions. To further purify and concentrate EVs, the combined fractions 7–10 were ultracentrifuged for 70 min at 110,000×*g* (average) at 4 °C. The resulting supernatant was discarded, and the pellet—containing the small tissue EVs—was resuspended in PBS containing PI/PS. Downstream experiments included nanoparticle tracking analysis (NTA), transmission electron microscopy (TEM), protein quantification by bicinchoninic acid (BCA) assay (Thermo Scientific #PI23227), Western blot analysis, and proteomics, for which the final EV pellet was resuspended in filtered PBS.

### NTA measurement

The size and number of particles in EV and 10k fractions were determined by ZetaView Nanoparticle Tracker (Particle Metrix GmBH, Meerbusch, Germany), and the corresponding ZetaView software (8.05.14 SP7). The instrument was calibrated with 100 nm diameter beads (Thermo Scientific, catalog #4310A). The samples were diluted in filtered PBS and injected into the viewing chamber with a syringe. Light scattering was recorded at 488 nm wavelength with the following settings: Temperature: 23 °C; camera sensitivity: 80.0; Shutter speed: 100; Focus: autofocus; frame rate: 30 frames per second. The analysis was conducted with the following parameters: minimum brightness 20, maximum particle size 1000 nM, minimum particle size 10 nM.

### Transmission electron microscopy

Freshly thawed preparation of EVs and 10k pellet were adsorbed onto glow-discharged ultrathin carbon coated copper grids of 400 mesh (EMS CF400-CU-UL) for 2 min. The grids were quickly rinsed in 1X Tris-buffered saline (TBS) three times. After rinsing, the grids were negatively stained with 1% Uranyl Acetate (UA) with 1% tylose in deionized water double-filtered with a 0.22 µm syringe filter. The grids were dried by blotting and imaged on a Hitachi 7600 TEM operating at 80 kV with an AMT XR80 CCD (8 megapixel). Ten images of three independent spinal cord EV samples were taken for each time point.

### Protein quantification and Western blot

Spinal cord tissue pellets obtained after the 300×*g* centrifugation step were resuspended in 1× RIPA lysis buffer (Cell Signaling Technology #9806) containing 1× PI/PS and incubated on ice for 30 min. Tissue was homogenized and sonicated on ice for 20 s. The homogenate was centrifuged at 10,000×*g* for 5 min, and the supernatant was collected. All other samples were lysed using RIPA buffer, sonicated for 20 s on ice, and spun to eliminate debris.

Protein concentration was determined by BCA protein assay kit according to the manufacturer’s protocol. Briefly, lysed samples were diluted, mixed with BCA working reagent in a 96-well plate, and incubated for 30 min at 37 °C. Absorbance was measured at 562 nm. A bovine serum albumin (BSA) standard curve was used to determine protein concentrations.

For SDS-PAGE, 6 µg samples diluted in Laemmli buffer (non-reducing) was loaded onto 4–20% Mini-PROTEAN® TGX™ precast protein gel (Cat# 4561096, Bio-Rad). After separation, proteins were transferred onto a methanol-activated 0.45 µm polyvinylidene difluoride membrane (88585, ThermoFisher). Membranes were blocked with freshly prepared intercept blocking buffer (927-66003, LI-COR) and probed with specific primary antibodies in intercept (TBS) blocking buffer overnight at 4 °C. Primary antibodies were anti-CD81 (1:1000, Novus Biologicals, NBP1-77039; 1:500, Santa Cruz, Sc-166028), anti-Flotillin1 (1:1000, BD Biosciences, 610820), and anti-tumor susceptibility gene 101 (TSG101, 1:1000, BD Biosciences, 612692). After incubation, membranes were washed with 0.01% TBST and further incubated with 1:20,000 of IRDye® fluorophore-labelled secondary antibodies (LICOR-926) for 30 min at RT. Membranes were imaged using the LI-COR Odyssey® M imaging system (LI-COR biosciences).

### Sample preparation and LC–MS/MS analysis

The sample preparation and liquid chromatography-tandem mass spectrometry (LC–MS/MS) analysis were conducted as described previously with minor modifications [22]. EVs were dried under vacuum and reconstituted in lysis buffer containing 8 M urea (Sigma Aldrich) and 50 mM triethylammonium bicarbonate (TEAB, Sigma Aldrich). Samples were reduced and alkylated in the presence of 10 mM tris(2-carboxyethyl) phosphine (TCEP, Sigma Aldrich) and 40 mM 2-chloroacetamide (CAA, Sigma Aldrich) at RT for 1 h. EV proteins were digested with Lys-C (Lysyl endopeptidase MS grade; Fujifilm Wako Pure Chemical Industries Co, Ltd) in a ratio of 1:100 (final concentration of 2 ng/µL) at 37 °C for 3 h. Further digestion was conducted by diluting the urea concentration to 2 M by adding three volumes of 50 mM TEAB followed by trypsin (sequencing grade modified trypsin; Promega) in a ratio of 1:50 (final concentration of 10 ng/µL) and incubating at 37 °C overnight. The resulting peptides were desalted using C18 StageTips (3 M Empore; 3 M) and labeled with 18-plex TMTpro reagents (Thermo Fisher Scientific) after sample randomization according to the manufacturer’s instruction. The labeled peptides were pre-fractionated by basic pH reversed-phase liquid chromatography into 96 fractions and concatenated into 24 fractions, which were vacuum-dried and reconstituted in 0.5% formic acid (FA, Thermo Fisher Scientific) for mass spectrometry. The Orbitrap Fusion Lumos Tribrid Mass Spectrometer (Thermo Fisher Scientific) coupled with an Ultimate 3000 RSLCnano nanoflow liquid chromatography system (Thermo Fisher Scientific) was used to analyze fractionated peptides. Peptides from each fraction were loaded onto a trap column (Acclaim PepMap 100, LC C18, 5 μm, 100 μm × 2 cm, nanoViper) at a flow rate of 8 μL/min and resolved at a flow rate of 0.3 μL/min by increasing the gradient of solvent B (0.1% FA in 95% ACN) to 28% on an analytical column (Easy-Spray PepMap RSLC C18, 2 μm, 75 μm × 50 cm) fitted onto an EASY-Spray ion source operated at a voltage of about 2.5 kV. The overall run time was 120 min. MS analysis was performed in data-dependent acquisition and “Top Speed” modes with 3 s per cycle. MS1 scan range for precursor ions was set to m/z 300 to 1800. MS1 and MS2 mass resolution was set to 120,000 and 50,000 at an m/z of 200. MS2 scans were acquired by fragmenting precursor ions using the higher-energy collisional dissociation (HCD) mode, which was set to 35% of normalized collision energy. The automatic gain controls for MS1 and MS2 were set to one million and 0.05 million ions, respectively. The maximum ion injection times for MS1 and MS2 were set to 50 and 100 ms, respectively. The precursor isolation window was set to 1.6 with 0.4 of offset. Dynamic exclusion was set to 30 s, and singly charged ions were rejected. Internal calibration was carried out using the lock mass option (m/z 445.12002) from ambient air.

### Database searching and peptide quantification

Database searches and statistical analysis were conducted as described previously with minor modifications [22]. Proteome Discoverer (version 2.4; Thermo Fisher Scientific) was used for quantitation and identification. The top ten peaks in each window of 100 Da were selected for database search during MS2 preprocessing. The MS/MS data were then searched using SEQUEST HT algorithms against a *Mus musculus* UniProt database that includes both Swiss-Prot and TrEMBL (released in January 2019 with 55,435 entries) with common contaminant proteins (115 entries). The precursor mass (MS1) tolerances were set to 10 ppm and 0.02 Da for precursor and fragment ions, respectively. Trypsin was used as the protease, allowing for a maximum of two missed cleavages. Carbamidomethylation (+ 57.02146 Da) of cysteine and TMTpro tags (+ 304.20715 Da) on lysine and peptide N termini were set as fixed modifications. Oxidation (+ 15.99492 Da) of methionine and acetylation (+ 42.01057 Da) on protein N termini was selected as a variable modification. Peptides and proteins were filtered at a 1% false discovery rate (FDR) at the peptide-spectrum match level using a percolator node and at the protein level using the protein FDR validator node, respectively. Both unique and razor peptides were used for peptide quantification. Protein groups were considered for peptide uniqueness. The co-isolation threshold was set to 50%, and the reporter ion abundance was computed based on signal-to-noise ratios. The missing intensity values were replaced with the minimum value, and the average reporter signal-to-noise threshold was set to 50. Protein grouping was performed with a strict parsimony principle to generate the final protein groups [23].

### Further bioinformatic and statistical analysis

**Fig. 1:**
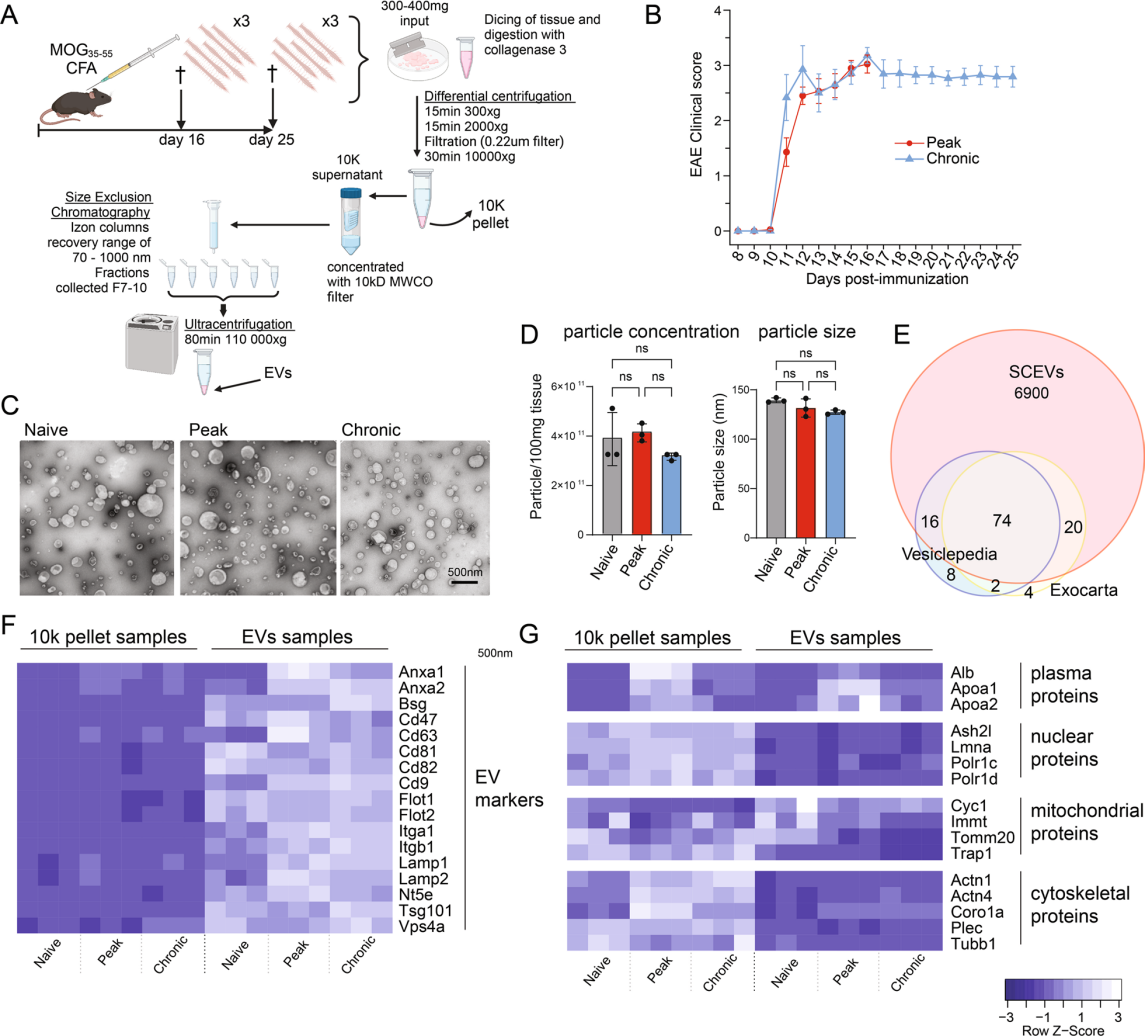
Workflow for spinal cord-derived EV separation and characterization. **a** The diagram depicting the EV separation method using size exclusion chromatography and ultracentrifugation (created using BioRender.com). **b** Clinical scores of mice at day 16 and day 25 showed no significant difference (n = 15 per time point). **c** Representative electron microscopy images of negatively stained EVs from spinal cords before EAE induction and at days 16 and 25 (ten images taken for three samples from each time point). **d** Particle concentration and size at the three time points obtained using NTA (bar graphs show the mean ± SD of n = 3 samples (pooled from 5 spinal cords) at each time point. Statistical significance was determined using one-way ANOVA and Turkey’s post-test. ns = not significant. **e** Number of the top 100 EV-enriched proteins, according to Vesiclepedia and Exocarta, present in the spinal cord EV proteome. **f** Heatmap showing the relative abundance of commonly used EV markers in spinal cord EV samples and 10k pellet samples (EV depleted particles) obtained as a byproduct of the EV isolation. **g** Heatmap showing the relative abundance of frequently described possible co-isolated proteins recovered from tissue during EV separation. (n = 3 for each time point and each sample type, i.e., EV and 10k)

In the following steps, data were reformatted in R (version 4.3.1, R Core Team). Uniprot IDs were matched to EntrezIDs and gene symbols using the AnnotationDbi package (version 1.62.2, [85]) and org.Mm.eg.db annotation (version 3.17.0, [86]). Principal component analysis was carried out using the stat package (version 3.6.2, R core team) and plotted using the autoplot function of ggplot2 (version 3.4.4). Proteins missing data points in any sample were excluded from analysis. Data were normalized using median centering and then log2 transformed. To compare overall protein content in EVs and 10k pellet, samples were normalized together (i.e., for Fig. 1). To compare changes at different time points, EV and 10k samples were normalized separately (all remaining analyses). We used LIMMA (version 3.56.2) [24, 25] for a three-group differential expression/abundance analysis comparing naïve, peak and chronic samples. We fitted a linear model [analysis of variance (ANOVA)] to the data and moderated our test statistics with empirical Bayesian methods using LIMMA. Volcano plots and heatmap were generated using ggplot2, ggrepel (version 0.9.3) and the heatmap.2 function of gplots (version 3.1.3.1). Enrichment analyses were carried out using clusterProfiler (version 4.8.2) [26]. The gene sets tested included the integrated biological pathway GO terms (GO.db version 3.17.0) [27], KEGG pathways [28] and Reactome (ReactomePA 1.44.0) [29] gene sets. Cut-offs were defined as < − 1.5/> 1.5-fold change and adjusted p-value < 0.05. Results were plotted using enrichplot (version 1.20.1) and ggplot2. For immune cell deconvolution analyses, mouse gene IDs were converted to human genes by orthology using Orthology.eg.db (version 3.17.0, Marc Carlson, 2023) and org.Hs.eg.db annotation (version 3.17.0, Marc Carlson, 2023). Deconvolution analyses were carried out with the online CIBERSORTx [30, 31] tool using the absolute mode, the integrated batch correction tool, and the integrated LM22 deconvolution matrix and Immunodeconv (version 2.1.0) [32] using the integrated Microenvironment Cell Populations-counter method. The LM22 deconvolution matrix uses the M1/M2/M0 macrophage polarization terminology to annotate cell types. We re-named these populations as homeostatic (for M0), pro-inflammatory (for M1) and alternatively activated (for M2). Statistical analysis of fold changes of immune populations was done in LIMMA as described above. For glia and neuron deconvolution analysis using BRETIGEA (version 1.0.3) [33], a singular value decomposition estimation method was used, and the number of markers for each cell type was set to 400. To further study the changes in synaptic protein subtypes, we queried the associated cellular compartment GO terms for terms associated with our subsets of interest. To compare changes in pre- and postsynaptic proteins, we queried the terms to include either “presynaptic” and not “postsynaptic” or “postsynaptic” but not “presynaptic”. To compare inhibitory and excitatory proteins in EVs, we queried either (1) both “GABA-ergic” and “inhibitory” or (2) both “glutamatergic” and “excitatory”. To further confirm the glial cell deconvolution, we compiled a list of unique glial EV markers using the overlap of three data sets: proteomics of cultured murine CNS cells [34], transcriptomics of murine CNS tissue [71], and proteomics of EVs from the supernatant of human iPSC-derived CNS cells [35]. Venn diagrams showing the dataset overlaps were generated using VennDiagramm (version 1.7.3) in R. For microglia, astrocytes, and neurons, our marker list was the overlap of all three datasets. For oligodendrocyte-lineage cells, the list was the overlap of the datasets from [34] and [36]. Statistical analyses of changes in cellular markers in supplementary Fig. S4 (in supplementary figures file) were carried out in GraphPad Prism 10 using a Friedman test with Dunn’s multiple comparisons. The graphs were also generated in Prism. The diagrams in Fig. 1 and Fig. S1 (in supplementary figures file) were created using Biorender.com, and figures were compiled using Adobe Illustrator 2023.

## Results

This study aimed to profile the changes in CNS tissue-derived EVs in EAE, a mouse model of multiple sclerosis, at the peak and chronic phase of disease, i.e. day 16 and 25 after induction, respectively.

### Spinal cord EV concentration and morphology are similar to healthy mice throughout EAE

EVs were separated from the whole spinal cords of naïve mice and mice with EAE using a previously published protocol [21] (Fig. 1A). EAE mice all developed hind limb paralysis with no significant difference in clinical scores recorded for mice taken down at day 16 or 25 post-immunization (Fig. 1B).

Nanoparticle tracking analysis (NTA) and transmission electron microscopy (TEM) were used to study physical characteristics of the EVs. There was no difference in the EV particle yield per tissue mass in naive mice compared to the EAE mice at the peak and chronic phase (Fig. 1D), nor was there in the size profile (Fig. 1D and S1A, in supplementary figures file). Further, in TEM EVs had a typical “cup-shaped” morphology (Fig. 1C), an artifact of the TEM fixation process.

Proteomics showed enrichment of EV markers including CD81, CD9, CD63, flotillin 1/2, annexin A1/A2 and tumor susceptibility gene 101 (TSG101) in the spinal cord EVs compared with the 10k pellet from the same mice (Fig. 1F). Western blots also confirmed enrichment of EV markers CD81, flotillin 1, and TSG101 (Fig. S1B-C, in supplementary figures file). Furthermore, EVs were depleted of nuclear proteins, cytoplasmic proteins, and most mitochondrial proteins: translocase of outer mitochondrial membrane 20 (TOMM20), TNF receptor associated protein 1 (TRAP1), and inner membrane mitochondrial protein (IMMT) (Fig. 1G), while cytochrome c1 (CYC1) was enriched in EVs. Blood plasma proteins albumin, apolipoprotein A1, and A2 were elevated at peak, when the BBB is most severely compromised, and associated with disease state (i.e., naïve, day 16, or day 25) rather than sample type (i.e., EV or 10k). 90% and 94% of the Vesiclepedia [37] and Exocarta [38] “top 100” EV proteins, respectively, were detected in EVs (Fig. 1E).

### EAE spinal cord EVs show disease-stage-dependent proteomic changes

**Fig. 2:**
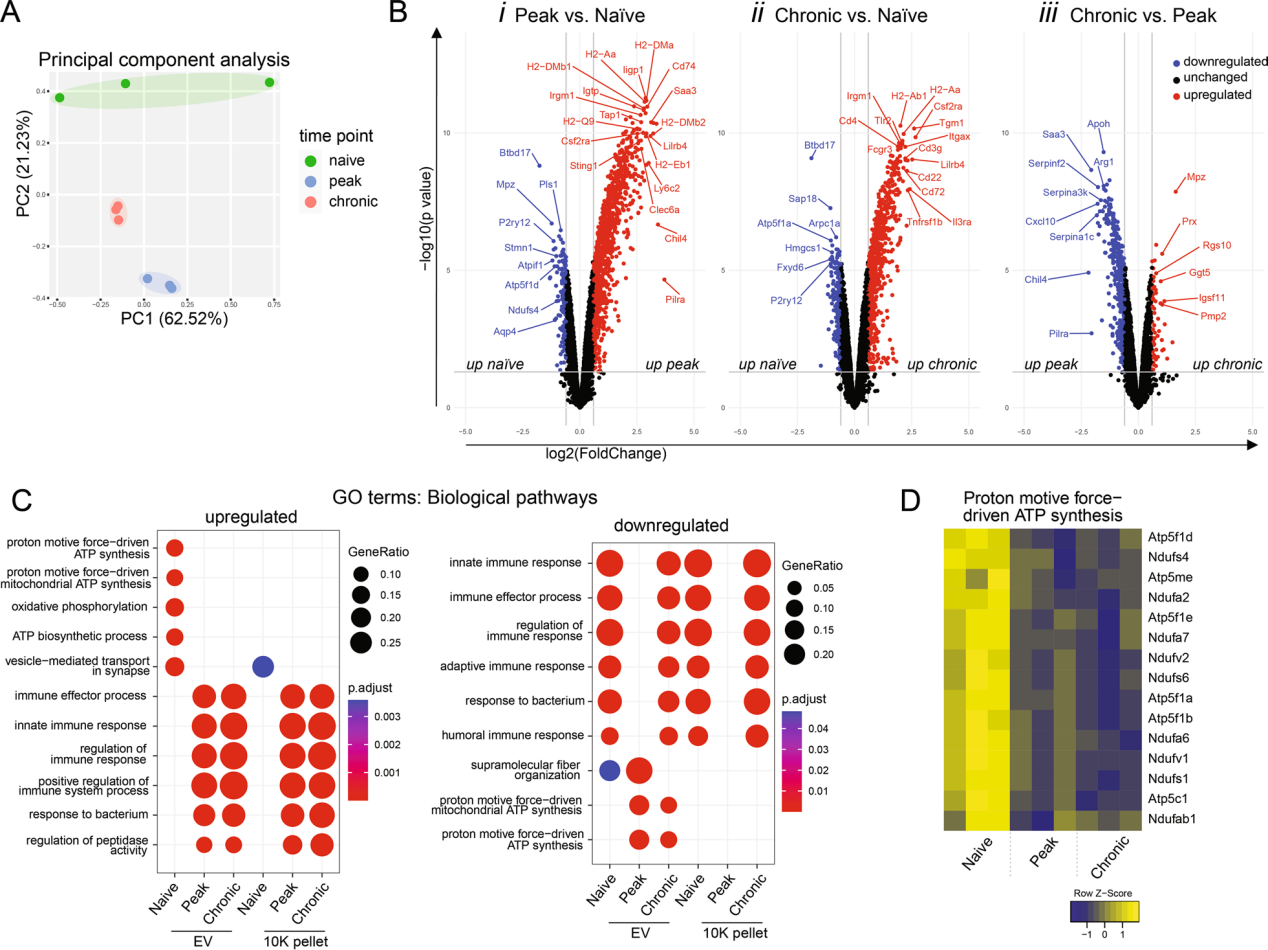
Proteomic changes in spinal cord EVs during EAE. **a** Principal component analysis using normalized abundances of proteins before EAE induction (green) and 16 (blue) and 25 (red) days after. **b** Volcano plots showing significantly up- (red) and downregulated (blue) proteins. Significance is defined by an adjusted p-value of < 0.05 and a < − 1.5 or > 1.5-fold change. Proteins are shown as gene symbols. **c** Dot plot of gene ontology pathway enrichment analysis showing the top upregulated and downregulated biological processes. The dot size represents the gene ratio, i.e., the identified proteins per gene set and the dot color represents the significance of the enrichment. **d** Heatmap showing the relative abundance of the proteins from the “proton motive force-driven ATP synthesis” GO term gene set. Proteins are shown as gene symbols [n = 3 (from 5 score-matched spinal cords) for each time point and each sample type, i.e., EV and 10k]

Proteomics detected 7010 proteins, of which 2823 were differentially abundant (adjusted p ≤ 0.02) in EAE at any time point in EVs, while only 1824 were differentially abundant in the 10k pellet. Principal Component Analysis (PCA) distinguished EV samples into three clusters representing naïve spinal cord EVs and the various EAE time points (Fig. 2A), indicating a dynamic alteration in tissue-derived EVs throughout the disease progression. (Fig. 2A).

Most of the top differentially abundant proteins (ranked by adjusted p-value) and pathways identified have known relevance in MS and EAE pathology. The most significantly dysregulated proteins at the peak of disease (vs. naïve) were all upregulated and were all inflammation-associated (Fig. 2Bi). Many of these were also among the top upregulated proteins (filtered for upregulated and ranked by p-value) at the chronic stage, although they were significantly less upregulated than at day 16 (Fig. 2Bii, 2Biii). The top 10 most significantly downregulated proteins (filtered for downregulated and ranked by p-value) at peak included myelin proteins, proteins associated with actin bundle formation, and glial markers—P2RY12 for homeostatic microglia and Btbd17 for astrocytes (Fig. 2Bi). Again, these changes were also observed in the chronic samples vs. naive, but to a lesser extent, except for Btbd17, which further decreased by day 25 (Fig. 2Bii, Biii).

Upon comparing the peak and chronic samples, the top 10 most significantly differentially abundant proteins were mostly downregulated at day 25 compared to day 16 (Fig. 2Biii). These included: plasma proteins including lipoproteins (ApoH, ApoA1), acute-phase proteins (haptoglobin), immunoglobins (Ighv4-1 immunoglobulin heavy variable 4-1), and coagulation proteins (prothrombin and α-2-antiplasmin); and inflammatory proteins promoting TH17 polarization (SAA3, Arg1). The only upregulated protein among the top 10 dysregulated proteins at day 25 compared with day 16 was the myelin protein MPZ. Other proteins, upregulated at the chronic time point compared with peak (i.e., the top 10 downregulated proteins) were: ceruloplasmin (Crb), which is involved in iron metabolism, and Schwann cell-associated regulators of peripheral nerve ensheathment, periaxin (Prx), Mpp6, and Naalad2.

Changes in individual proteins were reflected in hypergeometric gene set analysis performed with ClusterProfiler (Fig. 2C). On both days 16 and 25, immune response-related pathways were enriched. Compared with day 16, these inflammatory pathways were less significantly enriched at day 25 (among the top-down regulated pathways at the chronic stage). Proteins associated with the synaptic vesicle cycle, neurotransmitter regulation, and ATP synthesis were enriched in naïve samples. Interestingly, downregulation of ATP synthesis pathways was more prominent in EVs than in the 10k pellet. KEGG and Reactome pathway analysis confirmed the downregulation of synaptic vesicle and ATP synthesis-associated pathways and the upregulation of inflammatory pathways including the phagosome, complement, and coagulation cascade-associated pathways in both peak and chronic EAE (Fig. S2A-B in supplementary figures file). The proteins driving the changes in the “proton motive force driven ATP synthesis” pathway were NADH: ubiquinone oxidoreductase subunits (NDUFs) and ATPase subunits primarily associated with mitochondrial complexes I and V, respectively (Fig. 2D).

### Immune response-related proteins are strongly upregulated in EVs during EAE, especially at day 16, and the relative abundance of immune cell EVs changes throughout the course of EAE

Since immune response-related proteins were among the most significantly differentially abundant proteins in EVs at both day 16 and 25 compared with naïve mice, we further investigated the changes in these proteins. We assessed the proteins driving the enrichment of immune response-related pathways in the above-mentioned pathway analysis; investigated changes in the predicted immune cell type contribution using a bioinformatic cell deconvolution tool; and looked at changes in proteins that have been suggested as EV-associated biomarkers in people with MS (pwMS).

**Fig. 3:**
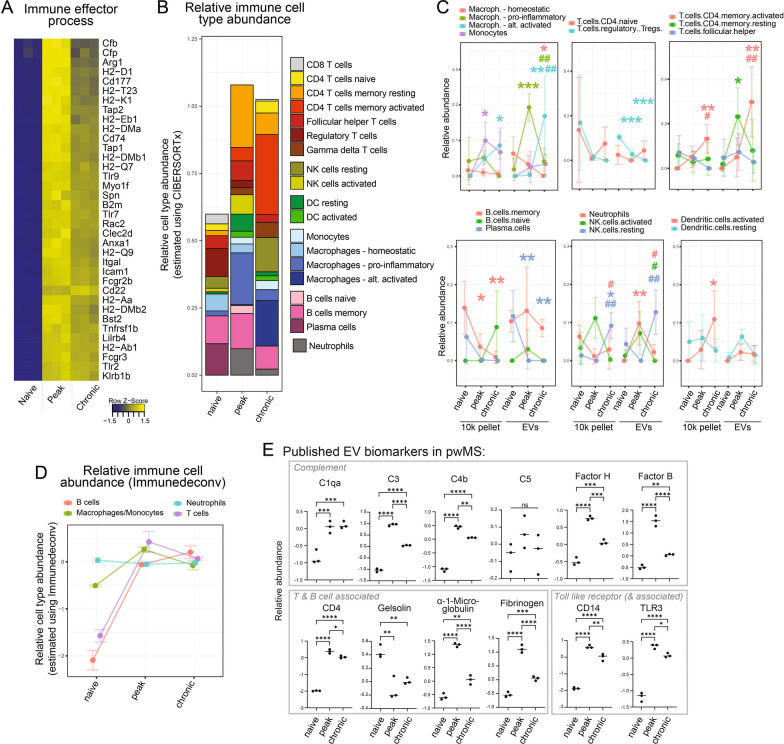
Immune response-related proteins and pathways in EVs during EAE. **a** Heatmap showing the relative abundance of the top proteins (adjusted p-value < 0.0001 and fold change of > 4 at day 16 or 25 compared to naïve samples) from the “immune effector processes” GO term. Proteins are shown as gene symbols. **b** Relative immune cell contribution to the spinal cord EV pool at different time points estimated using CIBERSORT cell deconvolution analysis. The graph shows mean predicted absolute contributions (SD and statistical analysis shown in **C**). Each bar represents a time point and each color a cell type. **c** Mean predicted cellular contribution (± SD; n = 3 samples) of distinct cell populations (colors) over time in the 10k pellet and EVs. Statistical analysis was performed using a linear model [analysis of variance (ANOVA)] in LIMMA. Stars (*) indicate comparisons of naïve to peak or chronic and hashes (^#^) represent comparisons of peak to chronic. P-values: * and ^#^p < 0.05, ** and ^##^p < 0.01, ***p < 0.001. **d** Mean relative immune cell contribution (± SD; n = 3 samples) to the spinal cord EV pool at the different time points using Immunedeconv cell deconvolution analysis. **e** Mean relative abundance (± SD; n = 3 samples) of selected published MS plasma EV biomarkers in our murine tissue EV samples. Significant differences are indicated as adjusted p-values per LIMMA fold change analysis. P-values: *p < 0.05, **p < 0.01, ***p < 0.001, ****p < 0.0001

The top 10 most significantly dysregulated proteins in EVs at peak of disease (vs naïve) were all immune-related proteins: antigen presentation-associated proteins (H2-DMa/b1, H2-Aa/b1, CD74) and immunity-related GTPases (Iigp1, Igtp, Irgm1)—all upregulated (Fig. 3A). Besides these, we found other immune-related proteins to be strongly upregulated by investigating the proteins driving the upregulation of the immune-related pathway. These included: other antigen presenting proteins (Tap1 and 2, B2m), pattern-recognition receptors (such as toll like receptors 2, 7, and 9), complement cascade proteins (complement C3, factor b, and factor properdin), and Fc gamma receptor (Fcgr2b and 3). All of these proteins were also upregulated at day 25 compared with naïve mice but were lower at this time point compared to day 16.

To assess possible changes in the cellular contribution to the tissue EV pool, we performed immune cell deconvolution analysis with CIBERSORTx (Fig. 3B and C). This tool uses single-cell expression data of 547 markers, of which we were able to detect 128, to deconvolute the abundance of immune cell types in a bulk sample. We excluded eosinophils and mast cells from the analysis because fewer than 4 out of the top 25 markers identifying these cells were expressed in our dataset. We found an increase in the relative abundance of immune cell-derived EVs after EAE induction: an increase of contributing T cells, NK cells, DCs, monocytes/macrophages, and neutrophils. An increase in T cell, B cell, and monocyte/macrophage sources was confirmed in another immune cell deconvolution analysis—Immunedeconv—(Fig. 3D) and when assessing selected markers (Fig. S3A in supplementary figures file). When assessing immune cell subsets, CIBERSORTx assigned a significantly reduced contribution of Tregs during EAE and a significantly increased contribution of activated CD4 memory T cell at day 25 compared with both naïve and day 16 (Fig. 3C). Interestingly, we also found a significant shift in the polarization of macrophages contributing to the EV pool. EV-associated pro-inflammatory macrophage markers significantly increased at day 16 but fell by day 25, while alternatively activated macrophage markers were increased at day 25 versus both naïve and day 16. There was a trend towards consistent reduction of homeostatic contribution, with a significant reduction at day 25 compared with naïve. The 10k pellet showed fewer significant changes in putative EV cell sources.

### Inflammatory EV biomarkers identified in people with MS are altered in EAE spinal cord EVs

Recent work on plasma-derived CNS- and immune cell-enriched EV biomarkers for MS has reported inflammation-associated proteins including T cell proteins (CD4 [16], gelsolin, and alpha1-microglobulin [17]), toll-like receptors (TLR3, TLR4 [8] and the TLR co-receptor CD14 [16]), coagulation cascade proteins (fibrinogen [18] and fibronectin [19]), and complement components (C1qa, C3, C4b, C5, Factor H and B [9]). To investigate the relevance of our findings in a preclinical model to human disease and determine how our findings in tissue might be reflected in plasma EVs, we examined the levels of these proteins in our samples (Fig. 3E). Most of the complement proteins, with the exception of C5, were significantly upregulated at days 16 and 25. C3, C4, Factor H, and B were significantly less abundant on day 25 than on day 16, while C1q remained unchanged on days 16 and 25. The T-cell associated proteins also followed the trend reported in plasma EVs of MS patients: CD4 and alpha1-microglobulin were upregulated at day 16 and to a lesser extent at day 25, and gelsolin was downregulated similarly at both days 16 and 25. CD14 and TLR3 were upregulated (significantly more on day 16 than on day 25), while TLR4 was not significantly altered in our study. Fibrinogen was upregulated as reported in plasma EVs, and fibronectin was downregulated at day 25 compared to day 16.

Besides plasma EVs, CSF-derived EVs have been studied in pwMS. We assessed the levels of proteins that have been shown to be unique to CSF EVs in pwMS compared to healthy controls [39] in our data set. Many of these proteins—in particular, inflammatory response-associated proteins (C3, S100a9, Ighg1, Apoe) and coagulation cascade proteins (fibrinogen and fibronectin)—were also upregulated with EAE compared to baseline in our study (Fig. S3B in the supplementary figures file).

### Glial and neuronal cell type contributions to tissue-derived EVs changes throughout EAE

**Fig. 4:**
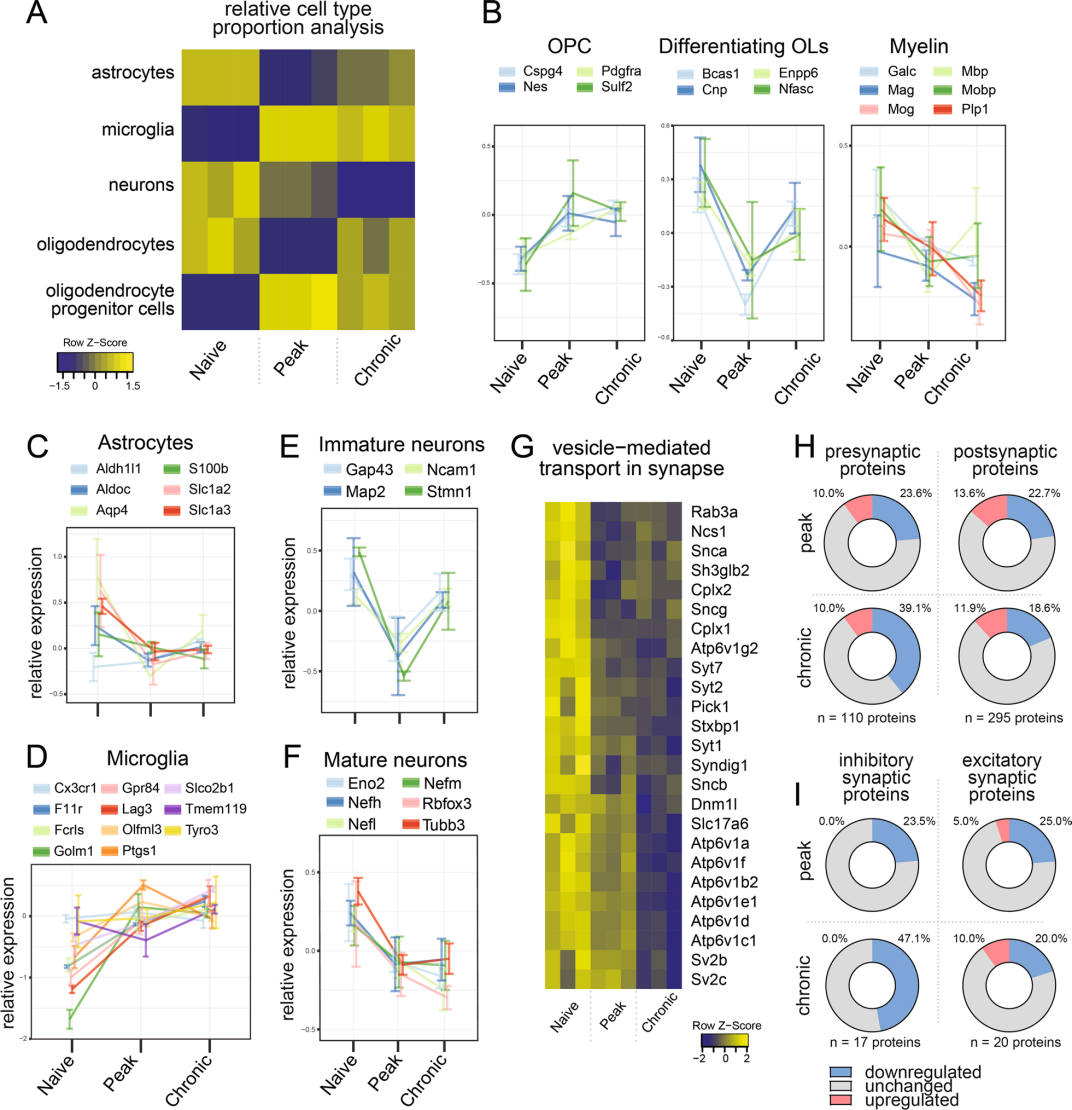
Glial and neuronal proteins and pathways in EVs during EAE. **a** Heatmap of the relative neuron and glial cell contribution to the spinal cord EV pool before and 16 and 15 days after EAE induction. The deconvolution analysis was carried out using BRETIGEA. Graphs showing mean relative abundance of selected **b** oligodendrocyte lineage, **c** astrocyte, **d** microglial, **e** immature neuronal, and **f** mature neuronal markers in EVs at the different time points (± SD; n = 3 samples). **g** Heatmap showing the relative abundance of the top proteins (filtered for adjusted p-value < 0.01 and fold change kept at < − 1.5/> 1.5) from the “vesicle-mediated transport in synapse” GO term enriched in our data. Proteins are shown as gene symbols. **h**, **i** Pie charts showing the change of pre- vs postsynaptic protein and inhibitory vs excitatory synapse protein abundance. The cellular compartment gene ontology terms associated with the proteins detected were filtered: for pre- vs postsynaptic analysis to include either “presynaptic” and not “postsynaptic” (110 identified proteins) or “postsynaptic” but not “presynaptic” (295 identified proteins); and for inhibitory vs excitatory to include either both “GABA-ergic” and “inhibitory” (17 identified proteins) or both “glutamatergic” and “excitatory” (20 identified proteins). Numbers on the pie chart represent the percentage significantly up- (red) or downregulated (blue) proteins of all identified proteins of the particular synaptic compartment or type (significance defined by p < 0.05 and fold change > 1.2/< − 1.2)

Relative glia and neuron cell type contribution to the EV pool was investigated using the Brain Cell Type Specific Gene Expression Analysis (BRETIGEA) [33], and confirmed with selected cell-specific markers. The glia and neuron deconvolution analysis was based on 400 markers per cell type: microglia, astrocyte, neuron, mature oligodendrocyte, and oligodendrocyte precursor cell (Fig. 4A). Decreased astrocytic, sharply upregulated microglial, continuously declining neuronal, transiently decreasing oligodendrocyte, and increased OL precursor cells (OPC) proteins were observed during EAE (Fig. 4A). This was further confirmed when assessing the change in glial cell and neuronal markers selected manually (Fig. 4B, C–F) and by the changes of cell-specific markers identified by selecting the overlapping proteins from proteomic analyses of EVs from human iPSC-derived CNS cell supernatants [35], proteomics of murine CNS cell cultures [34] and single-cell RNASeq of the murine CNS [13] (Fig. 4A–D).

We noted a difference in the dynamics of proteins associated with OPC differentiation versus the dynamics of myelin proteins (Fig. 4B). While myelin proteins continuously decreased, differentiation-associated proteins were less abundant at peak but partially recovered at day 25. Similarly, the dynamics of immature and mature neuronal markers in the EV pool markedly differed (Fig. 4E, F). The abundance of immature neuronal markers dropped at day 16 and recovered at day 25, while the relative contribution of mature neurons to the EV pool continuously decreased.

Although the predicted microglia contribution to the tissue EV pool in EAE was increased across all analyses, this was primarily driven by an increased abundance of inflammatory microglial markers (Fig. 4D), while homeostatic markers such as TMEM119 and P2RY12 were unchanged or significantly downregulated during EAE.

### EVs from EAE tissue contain lower levels of synaptic proteins with a particularly pronounced loss of presynaptic and inhibitory proteins

Next, we identified the proteins contributing to the reduced neuronal contribution observed in the cell deconvolution analysis and the perturbation of synaptic processes in the pathway analysis (Fig. 4G). Interestingly, we found numerous presynaptic proteins, including Stxbp1, Syt1, Syt7, Pick1, and Complexin I/II (cplx1/2), among the top dysregulated proteins in this pathway. Therefore, we next investigated if the loss in synaptic proteins shows distinct patterns, i.e., if there is a different dynamic in the change of pre- vs post-synaptic or inhibitory vs excitatory synaptic proteins. For this, we queried the cellular compartment gene ontology terms to include either “presynaptic” and not “postsynaptic” (n = 110) or “postsynaptic” but not “presynaptic” (n = 295) (Fig. 4H and S4F in supplementary figures file). We found a larger percentage of significantly downregulated presynaptic proteins (23.6% downregulated of all identified presynaptic proteins at day 16 and 39.1% at day 25) compared with postsynaptic proteins (22.7% at day 16 and 18.6% at day 25), with the largest difference at the day 25 time point. To compare the dysregulation of inhibitory and excitatory proteins in EVs we performed a gene ontology query for either both “GABA-ergic” and “inhibitory” (n = 17) or both “glutamatergic” and “excitatory” (n = 20) (Fig. 4I and S4F in supplementary figures file). We found a larger number of significantly downregulated inhibitory (23.5% downregulated of all identified inhibitory synaptic proteins at day 16 and 47.1% at day 25) than excitatory (25.0% at day 16 and 20.0% at day 25) synaptic proteins at day 25. We confirmed this by comparing inhibitory and excitatory proteins categorized according to a previously published inhibitory and excitatory synapse proteomics dataset [40], identifying 34 inhibitory and 141 excitatory proteins. 17.6% of inhibitory and 12.1% of excitatory proteins were reduced at day 16, while 35.3% of inhibitory and 18.4% of excitatory proteins were reduced at day 25 (Fig. S4E and S4G in supplementary figures file).

## Discussion

This study characterizes the dynamics of spinal cord-derived EVs during EAE, giving novel insights into possible roles of EVs in the pathophysiology of MS. We found marked changes in inflammatory, glial, and synaptic proteins and pathways, as well as a shift in the predicted contribution of various immune and glial cells types, corroborating that EVs can provide a snapshot of crucial disease processes such as CNS compartmentalized inflammation, re/de-myelination and synaptic pathology and might mediate them. We further found published inflammatory EV biomarkers identified in people with MS [8, 9, 16–19] to be altered in our EAE spinal cord EVs, suggesting an overlap of the pathological processes in EVs during EAE and MS and an overlap of EV proteomic changes in the CNS and periphery.

We chose spinal cord-derived EVs as the focus of this study because the spinal cord is an early and severely affected region in the EAE model and is clinically relevant to MS pathology. The major limitation of this choice was the small amount of spinal cord tissue compared with the less severely affected brain tissue per mouse. Despite pooling five spinal cords for three EAE score-matched biological batches for each time point, the protein output was low, limiting the number of possible downstream experiments. The selection of two time points, reflecting acute inflammation at day 16 and the initiation of resolution of neuroinflammation at day 25, offers a comprehensive view of EV changes across different phases of the disease.

Despite advances over the last decade, the separation, accurate characterization, and uniform classification of EVs remains challenging but constantly evolving. We followed a well-established, frequently used EV separation method involving differential centrifugation followed by SEC [21] and characterized the EVs according to the MISEV criteria [41]. We found a strong enrichment of known EV markers and a reduction of plasma, nuclear, cytosolic, and most mitochondrial proteins. A recent study comparing three CNS tissue EV separation protocols—SEC, sucrose gradient ultracentrifugation, and phosphatidylserine affinity capture—highlighted the speed and robustness of SEC, but also reported contamination with soluble proteins using this method [42]. However, our protocol differs from this publication in several steps: we carried out filtration with a smaller cut-off (0.22 µM vs 0.45 µM) and used higher centrifugation speeds to eliminate larger EVs—which reduces contamination and refines the studied EV subset. Indeed, in a direct comparison of 0.22 µM filtered to non-filtered EVs, it has been shown that 0.22 µM filtration resulted in a more homogenous population of EVs including exosomes and small microvesicles [43]. This aligns with the small EV size we detected with our method, and which others have obtained using this method [44]. To investigate if our observations were specific to or enriched in EVs, we compared the changes in the EV fraction to changes in the 10k pellet. In several analyses, we detected more pronounced changes in EVs, supporting interest in these particles providing a snapshot of ongoing pathological processes during EAE.

We characterized changes in the EV proteome using 18-plex TMT proteomics since this method allows for a small sample input and results in better detection of low abundance peptides and more robust quantification accuracy than traditional mass spectrometry methods. A limitation of this approach however is the lack of resolution of proteomic changes on a singular EV level or EV subpopulation level. New developments in cell-origin specific proteomics might help address this in future [45]. To nonetheless estimate how the relative contribution of different EV subpopulations changes throughout EAE we carried out deconvolution analysis using CIBERSORTx for immune cells and BRETIGEA for glial cells. One limitation of the use of these bioinformatic tools however is that they are based on cellular marker matrixes which might differ from markers expressed on EVs released from this cell type. Finally, when interpreting the changes in the predicted relative contribution of EV subpopulations, it also has to be noted that in the case of EVs (compared to cells) such changes can have manifold reasons including: a change in the number of cells that produce this EV subpopulation, a change in the secretory capacity of the cell type or change in the uptake or clearance of the EV subpopulation.

Our proteomics data identified several dysregulated proteins that play a role in the propagation and regulation of immune responses in EAE. At day 16 and, to a lesser extent, also at day 25, many of the top dysregulated proteins in EAE EVs compared to naïve EVs are related to MS-associated immune pathways. We noted a substantial increase in the abundance of antigen-presenting proteins in the spinal cord EVs from EAE mice. Whether this is due to an increased loading of antigen-presenting proteins onto EVs or an increase in infiltrating immune cells that produce antigen-presenting EVs is unclear. It has previously been shown that EVs can present antigen and activate leukocytes independent of their cell of origin either directly [46], with the help of cross-dressing bystander antigen-presenting cells [47], or through the transfer of MHC-mounted antigens onto acceptor cells [48]. It is tempting to speculate that the increase in antigen-presenting EVs might play a part in activating lymphocytes locally and in the periphery or bordering tissue since EVs are thought to cross the BBB [4]. Since we observe an increase in MHC class II, class I proteins and constituents of the immunoproteasome in EVs, this antigen presentation might occur through both direct- and cross-presentation mechanisms.

Another group of immune-related proteins upregulated in the EAE tissue-derived EVs were complement proteins. There are several reports of complement proteins in or possibly bound to EVs [9, 49]. Complement-containing EVs from adventitial fibroblasts induce proinflammatory responses in myeloid cells [49] and complement-containing circulating astrocyte-enriched EVs are neurotoxic and reduce neurite complexity [50]. In a previous study, our group found that plasma-derived astrocyte-enriched EVs from pwMS contained higher levels of complement components than healthy controls [9]. This suggests parallels between the changes we see in CNS-derived EVs in the EAE model and subsets of circulating EVs in pw﻿MS.

ATP synthesis and aerobic respiration-related proteins and pathways were among the most strongly downregulated in EAE EVs. EVs are known to shuttle glycolytic enzymes and produce ATP [51, 52]. In the CNS, Neuronal stem cell (NSC) EVs have been shown to contain several ATPases we found to be reduced in EAE in our study (Atp5f1d and a). The transfer of intact mitochondria to phagocytes through these NSC-EVs prevented the pro-inflammatory metabolic activation of phagocytes in vitro and in EAE [53]. Further, astrocyte-derived EVs, containing mitochondria, are reportedly shuttled to neurons and promote their survival [54]. In EAE, axonal mitochondria are reduced in number, less mobile, and show reduced complex 1 activity [55]. These signs of mitochondrial dysfunction are detected as early as day 15 [56]. This aligns with our observation of reduced complex 1 associated proteins in the EAE EVs, supporting the hypothesis that EVs may mirror changes occurring in cellular mitochondria and perhaps the disruption of homeostatic metabolic cues transferred by EVs may promote neurodegeneration and inflammation in EAE. In future, targeting these metabolic changes in EVs or restoring metabolic enzymes in EVs might be interesting therapeutic approaches in EAE and MS.

Besides inflammatory responses, we also detected marked changes in glial cell-derived EV proteins. Glia-specific cell deconvolution analysis showed that astrocyte and mature oligodendrocyte proteins are decreased in EAE EVs, while microglial and oligodendrocyte precursor proteins are upregulated. Demyelinating lesions with extensive loss of myelin and mature oligodendrocytes are hallmarks of both MS and EAE. In vitro, both murine and human mature oligodendrocytes have been shown to release myelin-containing EVs [7, 57]. In EAE spinal cord EVs the abundance of many myelin proteins was reduced at both day 16 and 25 post-immunization, with lower levels at the chronic time point, likely reflecting the loss of oligodendrocytes. Interestingly, two peripheral myelin proteins—MPZ and PMP2—and regulators of nerve ensheathment—Prx, Mpp6, and Naalad2—[58–60], were among the top upregulated proteins at day 25 compared to day 16. These proteins are predominantly found in Schwann cells from the peripheral nervous system (PNS). In mouse models of demyelination [61] and more recently also in spinal cord and cerebellar MS lesions [62] it has been shown that Schwann cells can migrate to demyelinated areas and in rare contexts contribute to remyelination. The PNS myelin protein and ensheathment regulator containing EVs in EAE may be an indicator of the role of Schwann cell remyelination in the CNS at day 25. Alongside the changes in myelin protein, we found an increase in proteins associated with OPC differentiation, likely reflecting the induction of this reparative process. The functional relevance of the changes in OL-derived proteins in the EAE tissue EV pool remains unclear. In the literature OL-EVs from culture supernatants have been shown to promote axonal health and function [63, 64]. Further OL-EVs injected into EAE mice suppress disease by inducing immunosuppressive monocytes and reducing autoreactive T cells [7]. However accumulating OL-EVs containing PLP, MAG, and MOG might also exacerbate disease, as they have been shown to block OPC differentiation in vitro [65].

Microglial and astrocytic proteins were also altered throughout EAE. Homeostatic microglial markers were among the top downregulated markers at both day 16 and 25, while the relative predicted microglial contribution using BRETIGEA [33] showed a sharp increase in microglial contribution. This aligns with previous studies that have reported a higher number of microglia- (and macrophage-) derived EVs in the CSF during EAE and in pwMS [10]. The increase could be explained by a higher EV secretory capacity of microglia in response to inflammatory stimuli, such as ATP or pro-inflammatory cytokines in EAE. In vitro ATP treatment of microglia induces their release of EVs, that contain inflammatory proteins and promote the induction of neurotoxic astrocytes [66]. Similarly, treatment of microglia with IL-1β, TNF-α, and IFN-ɣ induces their release of pro-inflammatory EVs that block OPC differentiation in vitro and remyelination in vivo through indirect effects on astrocytes [67]. Complementing these findings, homeostatic and regenerative microglia, induced by IL-4 or co-culturing with immunosuppressive mesenchymal stem cells, produce EVs that dampen inflammation and promote remyelination and synaptic function [15, 67]. Our finding of reduced homeostatic microglial markers, such as TMEM119 and P2YR12, in tissue EVs could therefore also contribute to a disruption of these functions at day 16 and 25 of EAE. A reduction of these homeostatic markers in EVs has also been reported in humans, in CNS tissue-derived EVs from Alzheimer’s patients [68].

With respect to astrocytic EVs, we noted a reduction in their relative contribution to the tissue EV pool, using cell deconvolution analysis. Possible explanations for this could be reduced production of astrocyte-derived EVs or faster CNS clearance or uptake. The former seems less likely considering previous studies that have shown increased astrocytic EV production in vitro in response to inflammatory stimuli [69, 70]. Interestingly, supporting the latter, Dickens et al. have shown that neuroinflammation-induced astrocytic EVs rapidly cross the BBB and regulate the peripheral immune response to CNS inflammation [70]. Besides clearance across the BBB, astrocyte-derived EVs might also be depleted from the tissue EV pool by cellular uptake during neuroinflammation. Supporting this, EVs produced by reactive human astrocytes in vitro show significantly higher uptake into neurons than resting astrocyte EVs [71]. Synaptic pathology and neurodegeneration are hallmarks of MS and EAE, and were detectable in the proteome of the EVs in our study. Synaptic vesicle and neurotransmitter regulation-related pathways were among the top dysregulated GO terms downregulated at both day 16 and 25 of EAE and the neuronal contribution to the EV pool was reduced in the BRETIGEA deconvolution analysis. Our results suggest that in EAE neuronal EV release is reduced (or neuronal EVs are more rapidly cleared) and they contain lower levels of synaptic proteins. Based on the physiological function of neuronal EVs [73–79], this might contribute to the synaptic pathology and neurodegeneration in the model. The more pronounced reduction of inhibitory synapse-associated proteins and post-synaptic proteins compared to excitatory and presynaptic proteins, is particularly noteworthy, since these imbalances have been described as a pattern of synaptic pathology in EAE and MS: Inhibitory networks are disrupted earlier, and more pronounced in EAE [80], MS and focal demyelination models [81, 82], compared to excitatory networks. Interestingly, our findings also align with EV biomarker studies in pwMS. Previously, we detected lower levels of the synaptic proteins in circulating neuronal EVs of pwMS compared to healthy controls [9]. Further, a recent study by another group showed reduced circulating levels of neuronal EVs in pwMS compared to healthy controls [17], though in our earlier study with a comparable cohort size, we did not see any differences [9]. Alongside the loss in mature neuronal markers, we observed a loss in immature neuronal markers in the EAE EVs, the latter however normalized at day 25, indicating an increased number of neuronal progenitors or an increase in their secretory activity.

Besides providing insight into the pathological changes in EVs during EAE, our study is also a resource for future studies investigating biomarkers or developing EV-based or -targeted therapies in EAE and ultimately MS. One possible approach for EV-targeted therapies is to inhibit EV release, with the aim to prevent the spread of pathological signals contained in them. This has shown some success in preclinical models of acute brain injury and Alzheimer’s disease [83, 84]. Further knockout of A-SMase, which is required for the shedding of microvesicles, has also been shown to be protective in EAE. Yet these are preventative studies and their protective effect might primarily be explained through the dampening of EVs’ contribution to neuroinflammation. Our data shows a shift of the EV proteome through the course of EAE from predominantly inflammatory to attempted remyelinating and regenerating proteins, including an increase of OPC differentiation-associated, neuronal progenitor marker proteins and alternatively activated macrophage markers at day 25 compared to day 16. This suggests that continuous blocking of EV release, though beneficial in early inflammatory disease stages, might worsen the outcome later by inhibiting beneficial EV signaling during the resolution of the neuroinflammation and repair. Indeed, there is evolving literature on the protective effects of EVs subsets in neuroinflammation [7]. In future studies, it will be interesting to investigate the functional relevance and factors regulating the release of the OPC, newly-formed OLs or neuronal progenitor protein containing EVs we characterized in our study.

Our study also provides insights into how plasma and CSF EV biomarkers might reflect changes in CNS tissue EVs and whether changes noted in EVs during EAE reflect changes in EVs in pwMS. Overall, we identified several changes in the EAE tissue EV proteome aligned with changes in EV biomarkers in MS including changes in complement proteins, T-cell and B-cell associated proteins, and TLRs. We and others have found increased complement component levels in plasma astrocyte-enriched EVs [9] and CSF EVs [39] in pwMS, which coincides with what we now detected in EAE tissue EVs. Which cell types drive the changes in complement we detect in our spinal cord EVs however remains unclear but could be investigated in future in light of the new developments in cell-origin specific proteomics [45]. T- and B-cell-derived plasma EV biomarkers include CD4, alpha1-microglobulin (Ambp), fibrinogen (Fgb), and gelsolin (Gsn) content [17]. In both plasma EVs from pwMS (compared to healthy controls) and in our EAE spinal cord EVs (compared to naïve mice) Gsn was reduced, and Ambp and fibrinogen were increased. Fibrinogen and fibronectin have also been detected in MS CSF EVs (but not in healthy control CSF EVs) [39] and, interestingly, fibrinogen-containing EVs have been shown to induce CNS-reactive T cells in EAE [18]. CD4+ plasma EV numbers are increased in active compared to stable MS [17] and in RRMS compared to healthy controls [16]. In EAE tissue EV CD4 levels were elevated at peak of disease compared to naïve mice and showed some normalization at the chronic time point compared to peak. The changes in plasma EV TLR3 and CD14 levels in pwMS [8, 16] were also noted in EAE EVs in our study [8, 16]. Taken together, the above-described overlaps in changes in tissue EVs in EAE and plasma/CSF EVs in MS suggest that there might be commonalities in the role of EVs in EAE and MS and that some changes observed in the CNS tissue EVs might also be observed in the periphery. In the future, obtaining side-by-side data sets of plasma and tissue EVs will give further insights into which EVs are crossing the BBB at different disease stages and whether the BBB shows a changed selectivity to specific EV subpopulations throughout disease. This might further help identify new circulating CNS-derived EV biomarkers.

## Conclusion

Spinal cord-derived EVs showed dynamic changes in inflammatory, glial and synaptic proteins and pathways, as well a shift in the predicted contribution of immune and glial cell types through the course of EAE. Our findings provide insight into EV-mediated cellular communication during EAE disease processes, such as such as CNS compartmentalized inflammation, re/de-myelination, and synaptic pathology. Further, our findings can be used as a resource to query proteins of interest in EV biomarkers or EV-targeted therapeutic studies.

### Supplementary Information

Supplementary Material 1. Supplementary figures S1–S4 with legends.

Supplementary Material 2. Proteomics dataset for EV and 10k samples.

## Data Availability

The datasets supporting the conclusions of this article are included within the article and its additional files.

## Abbreviations

ANOVA: Analysis of variance
ATP: Adenosine triphosphate
BBB: Blood brain barrier
BCA: Pierce bicinchoninic acid assay
BRETIGEA: BRain cEll Type specIfic Gene Expression Analysis
CFA: Complete Freund’s adjuvant
CIBERSORTx: Cell-type identification by estimating relative subsets of RNA transcripts
CNS: Central nervous system
EAE: Experimental autoimmune encephalomyelitis
EVs: Extracellular vesicles
FDR: False discovery rate
GO: Gene ontology
iPSC: Induced pluripotent stem cells
kDa: Kilodalton
KEGG: Kyoto Encyclopedia of Genes and Genomes
LIMMA: Linear models for microarray data
MHC: Major histocompatibility complex
MS: Multiple sclerosis
NTA: Nano-tracking analysis
OL: Oligodendrocyte
OPC: Oligodendrocyte precursor cells
PBS: Phosphate buffered saline
PI/PS: Protease inhibitor and PhosphoSTOP
PNS: Peripheral nervous system
PRR: Pattern recognition receptor
pwMS: People with MS
SEC: Size exclusion chromatography
TEM: Transmission electron microscopy
TLR: Toll-like receptor
TMT: Tandem mass tags
