# Supplementary material for: Characterization of spinal cord tissue-derived extracellular vesicles in neuroinflammation
Source: J Neuroinflammation. 2024 Jun 8;21:154. doi: 10.1186/s12974-024-03147-y (PMC11162576; doi:10.1186/s12974-024-03147-y)
Supplement: Supplementary file 1 — Supplementary Material 1. Supplementary figures S1–S4 with legends. [file 12974_2024_3147_MOESM1_ESM.pdf]

Supplementary Figures File

Manuscript: Characterization of spinal cord tissue-derived extracellular vesicles in neuroinflammation

Authors*: Larissa Jank, Ajay Kesharwani, Taekyung Ryu, Deepika Joshi, Dimitrios C. Ladakis, Matthew D. Smith, Saumitra Singh, Tanina Arab, Kenneth W Witwer, Peter A. Calabresi, Chan-Hyun Na, Pavan Bhargava*

<u>Figure S1:</u>

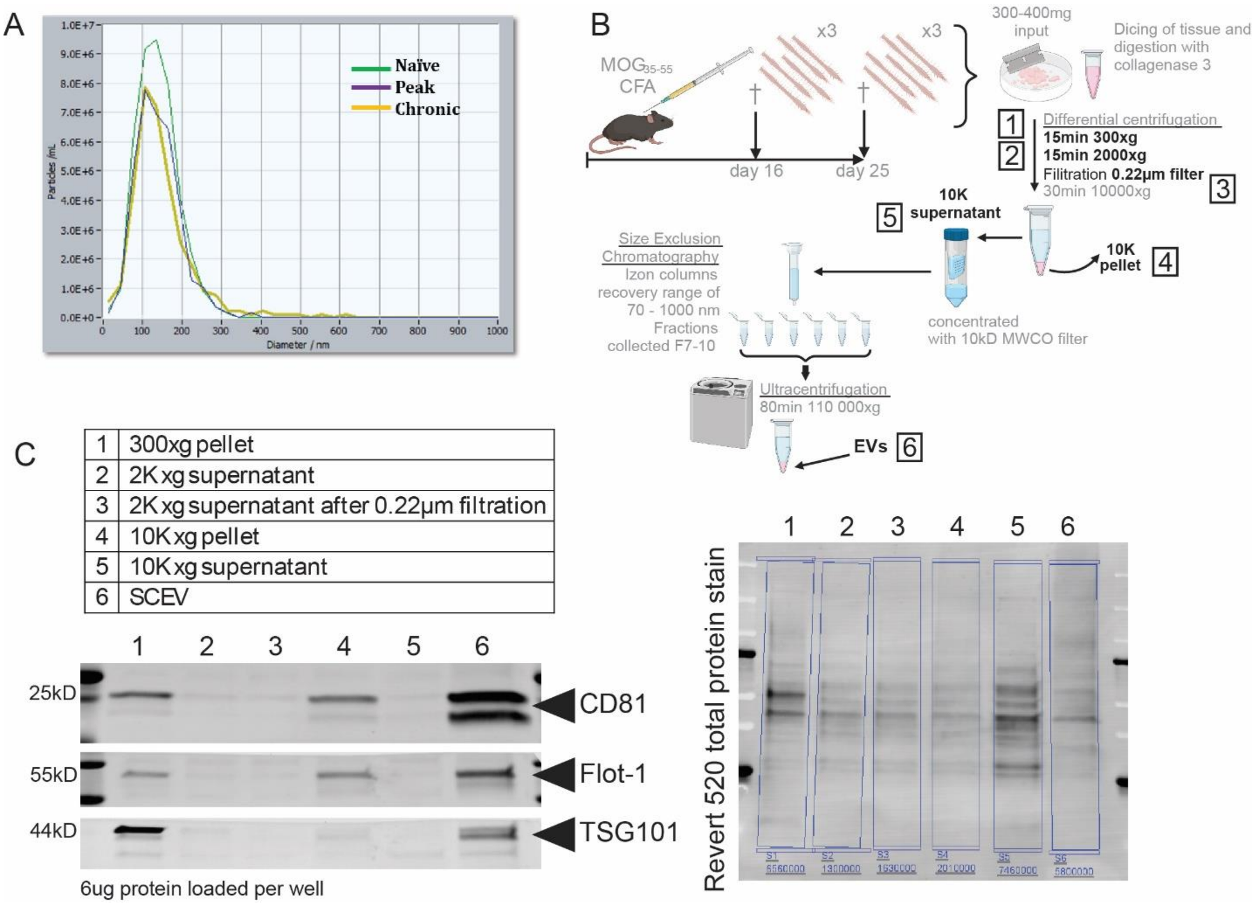

Figure S1: Details on the workflow for spinal cord-derived EV separation and characterization. **a** Histograms showing the distribution of EV diameter at the different time points **b** Diagram depicting the EV separation method and intermediate samples collected and characterized. **c** Table with intermediate samples collected during EV isolation and western blot showing the abundance of EV markers (CD81, Flot-1 and TSG101) and total proteins (Revert 520) in these samples (n=1 representative sample [from 5 score-matched spinal cords] for each time point)

Figure S2:

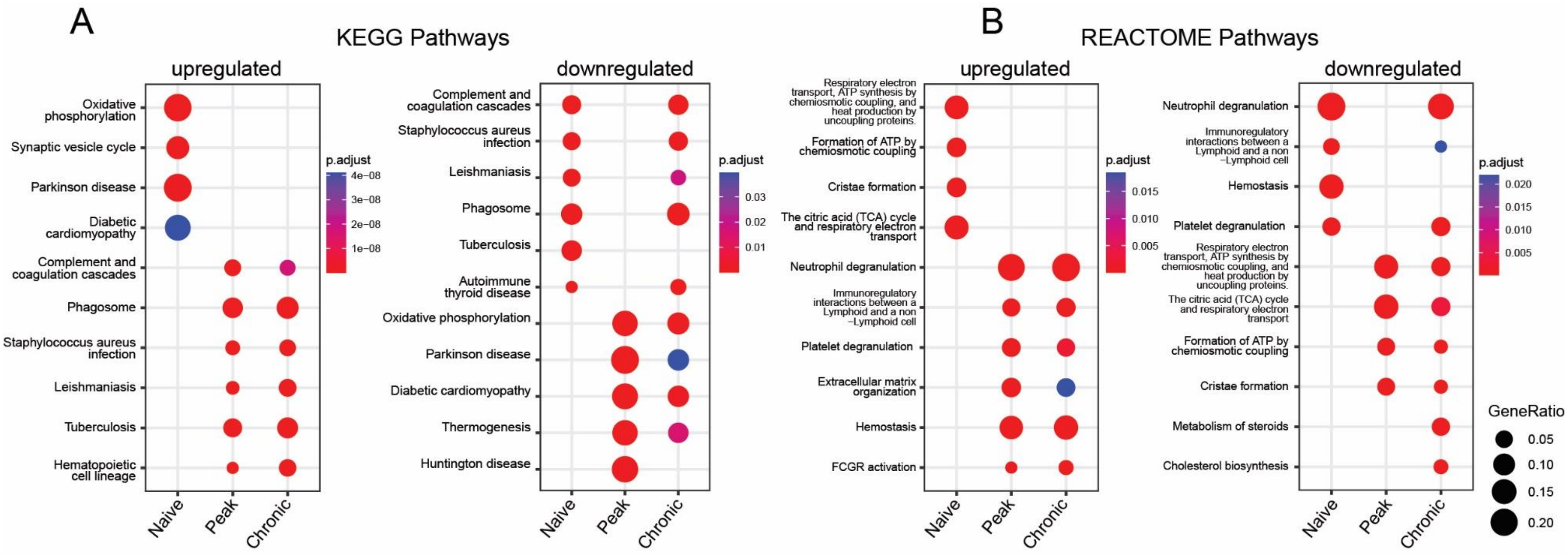

Figure S2: Further gene set enrichment analyses. **a** Dot plot of KEGG pathway enrichment analysis showing the top upregulated and downregulated biological processes. **b** Dot plot of REACTOME pathway enrichment analysis. The dot size represents the gene ratio, i.e., the identified proteins per gene set and the dot color represents the significance of the enrichment. (n=3 [from 5 score-matched spinal cords] for each time point and each sample type, i.e., EV and 10K)

Figure S3:

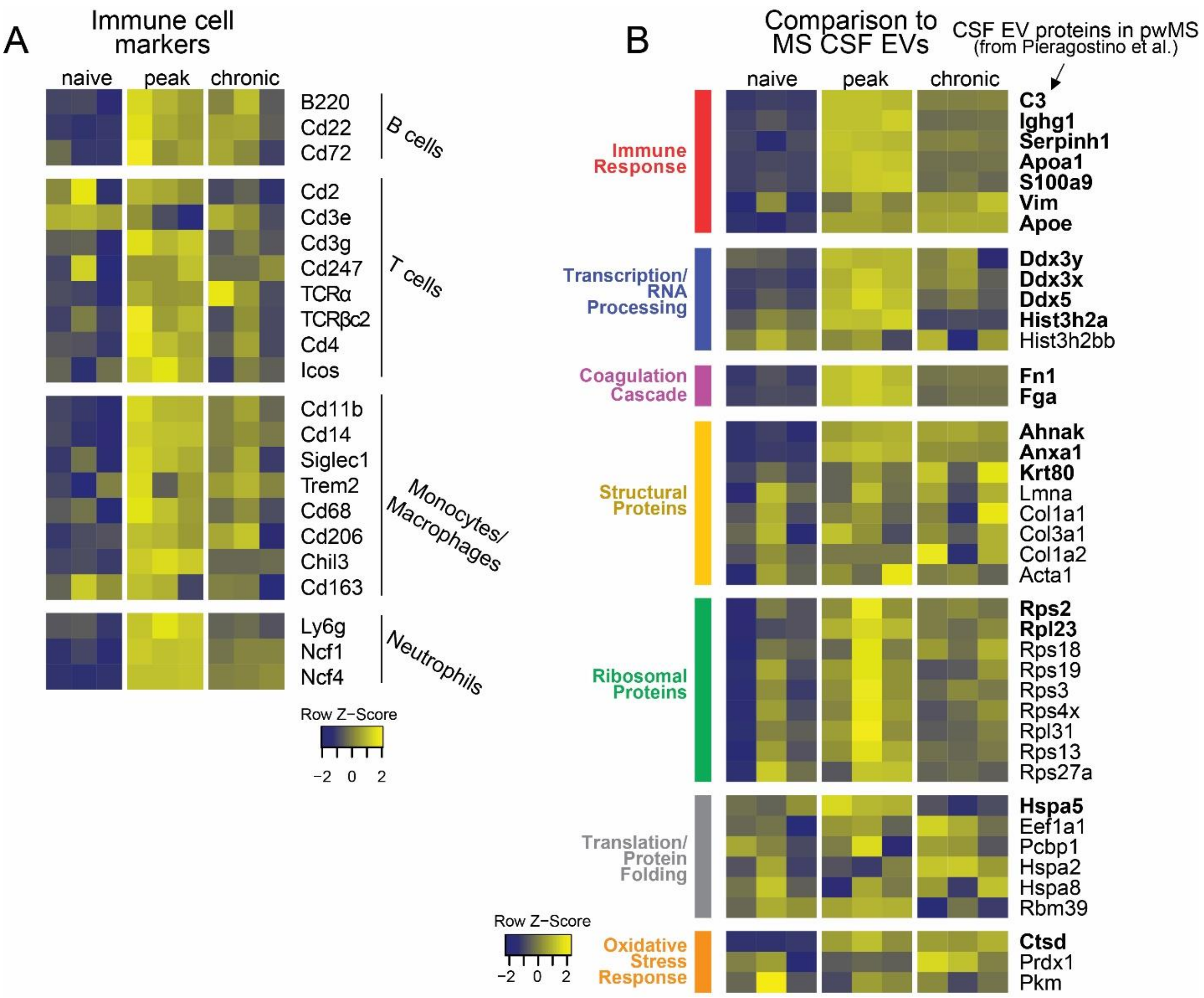

Figure S3: Confirming the immune cell deconvolution analysis results with selective markers. **a** Heatmap showing the relative abundance of immune cell markers in EVs at the three different time points. **b** Heatmap showing the relative abundance of proteins uniquely detected in CSF EVs of pwMS (but not in healthy controls, [39]) in our EAE samples at the three different time points. Significantly (adjusted p-value <0.05 for naïve vs peak or naïve vs chronic) altered proteins are highlighted in bold. (For both heatmaps n=3 [from 5 score-matched spinal cords] for each time point and each sample type, i.e., EV and 10K)

Figure S4:

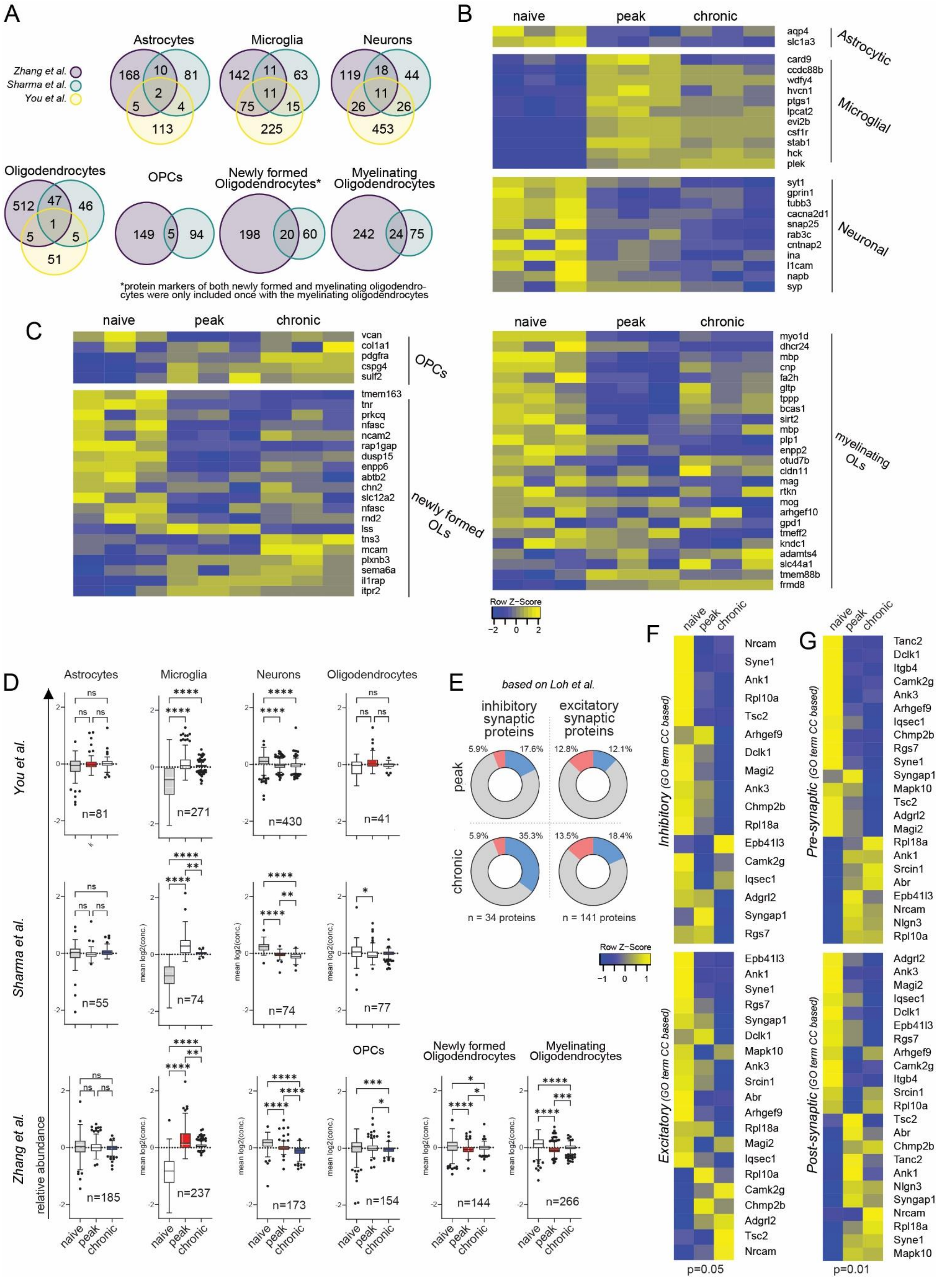
A
Zhang et al.
Sharma et al.
You et al.
Astrocytes
Microglia
Neurons
Oligodendrocytes
OPCs
Newly formed Oligodendrocytes*
Myelinating Oligodendrocytes
*protein markers of both newly formed and myelinating oligodendrocytes were only included once with the myelinating oligodendrocytes
B
naive
peak
chronic
Astrocytic
Microglial
Neuronal
C
OPCs
newly formed OLs
myelinating OLs
Row Z-Score
D
relative abundance
n=81
n=271
n=430
n=41
n=55
n=74
n=74
n=77
n=185
n=237
n=173
n=154
n=144
n=266
E
based on Loh et al.
inhibitory synaptic proteins
excitatory synaptic proteins
n = 34 proteins
n = 141 proteins
F
Inhibitory (GO term CC based)
Excitatory (GO term CC based)
p=0.05
G
Pre-synaptic (GO term CC based)
Post-synaptic (GO term CC based)
p=0.01

Figure S4: Confirming the glia and neuron cell deconvolution analysis results with selective markers. **a** Venn diagrams showing the overlaps of glia and neuron markers identified by [36], [34], and [35] **b-c** Heatmap showing the relative abundance (± SD) of selected glia and neuron cell markers in EVs at the three different time points **d** Mean relative abundance of all identified glia and neuron markers identified by [71], [89] and [93]. The number of plotted markers indicated with n. Friedman test with Dunn's multiple comparison test; P values: * $p < 0.05$, ** $p < 0.01$, *** $p < 0.001$, **** $p < 0.0001$. **e** Pie charts showing the change of inhibitory vs excitatory synapse protein abundance based on markers previously identified by [78]. Numbers on the pie chart represent the percentage of significantly up- (red) or downregulated (blue) proteins of all identified markers of the particular synaptic type (significance defined by $p < 0.05$ and fold change >1.2/<-1.2). **f-g** Heat map showing the mean relative abundances of the most significantly up- or downregulated pre- vs postsynaptic proteins and inhibitory vs excitatory synapse proteins (identified by filtering GO CC terms) at the different time points.
